# Supplementary material for: Involvement of the AMPK Pathways in Muscle Development Disparities across Genders in Muscovy Ducks
Source: Int J Mol Sci. 2024 Sep 21;25(18):10132. doi: 10.3390/ijms251810132 (PMC11432404; doi:10.3390/ijms251810132)
Supplement: Supplementary file 1 [file ijms-25-10132-s001.zip › ijms-3159431-supplementary.pdf]

Table S1. Data statistics of growth traits

Growth traits (mm)	Body weight （kg）	Body depth	Half-immersion length	Keel length	Chest width	Chest depth	Hip bone width
Female	2.28±0.081	263.33±3.98	294.67±3.22	191.33±1.65	128.28±1.33	80.46±1.33	78.33±1.24
Male	3.96±0.145	322.67±3.96	367.67±3.12	221.67±2.27	148.04±1.32	85.58±4.40	91.89±1.39
P-value	< 0.01	< 0.01	< 0.01	<0.01	<0.01	0.32	<0.01

Female,The female Muscoy ducks group. Male,The male Muscoy ducks group. *P*-value < 0.01,more pronounced significant difference.

Supplemental Table S2 Sequencing Data and Quality Assessment

Sample	Raw Reads	Bases（Gb）	GC(%)	Q20(%)	Q30(%)	Avg. Quality
Male1	47,161,350	7.074	50.73	98.82	96.58	36.675
Male2	42,276,254	6.341	50.66	98.775	96.445	36.645
Male3	51,209,992	7.681	52.17	98.77	96.47	36.655
Fmale1	39,965,644	5.995	51.505	98.84	96.61	36.67
Fmale2	43,330,474	6.5	51.84	98.7	96.245	36.585
Fmale3	42,354,460	6.353	50.925	98.6	95.9	36.49

Sample,Sample Name. Raw Reads:The number of raw data reads. Bases:The size of the data, measured in units of 1G base pairs. GC(%),The proportion of GC in the original DNA sequence. Q20:The proportion of base qualities greater than 20 in the raw data. Q30,The proportion of base qualities greater than 30 in the raw data. Avg.Quality:Average quality value.

Supplemental Table S3 Comparing with the mallard duck reference genome

Sample	Total reads after filtered	Mapped on reference	Unmapped	Multi-mapped	Non-splice reads	Splice reads
M1	45,556,202	29,623,303(65.03%)	15,932,899(34.97%)	2,483,776(5.45%)	13,156,508(28.88%)	13,983,019(30.69%)
M2	40,806,380	25,735,750(63.07%)	15,070,630(36.93%)	1,886,659(4.62%)	11,761,312(28.82%)	12,087,779(29.62%)
M3	49,454,618	28,838,183(58.31%)	20,616,435(41.69%)	2,232,160(4.51%)	12,675,297(25.63%)	13,930,726(28.17%)
Fm1	38,623,324	22,680,169(58.72%)	15,943,155(41.28%)	2,058,347(5.33%)	9,849,119(25.5%)	10,772,703(27.89%)
Fm2	41,782,326	24,538,229(58.73%)	17,244,097(41.27%)	2,100,941(5.03%)	10,924,576(26.15%)	11,512,712(27.55%)
Fm3	40,748,864	23,052,534(56.57%)	17,696,330(43.43%)	1,636,609(4.02%)	10,606,677(26.03%)	10,809,248(26.53%)

Sample,Sample Name. Total reads after filtered,The total number of filtered reads. Mapped on reference,The number of reads aligned to the reference genome. Unmapped,The number of reads that do not align to the reference genome. Multi-mapped,The number of reads aligned to multiple locations. Non-splice reads,Number of unspliced read alignment. Splice reads,Number of read alignments by splicing comparison.

Supplemental Table S4 Differentially down-regulated Genes in Muscovy ducks' muscle tissue

Name	Description	log2FC	trend
evm.TU.chr1.1885	cyclin-dependent kinase inhibitor 1-like	-5.111231556	DOWN
evm.TU.chr30.108	HOXC11; homeobox C11	-4.760593164	DOWN
evm.TU.chr19.434	myosin heavy chain, skeletal muscle, adult	-4.713536118	DOWN
evm.TU.chr11.507	ERC2; ELKS/RAB6-interacting/CAST family member 2	-3.992663903	DOWN
evm.TU.chr1.2122	PVALB; parvalbumin	-3.369544514	DOWN
evm.TU.chr7.454	SGPL1; sphingosine-1-phosphate lyase 1	-3.224852618	DOWN
evm.TU.chr3.145	OTOR; otoraplin	-2.889278429	DOWN
evm.TU.chr2.602	CMBL; carboxymethylenebutenolidase homolog	-2.772413675	DOWN
evm.TU.Contig580_pilon.2	JMY; junction mediating and regulatory protein, p53 cofactor	-2.743600436	DOWN
evm.TU.chr30.109	HOXC10; homeobox C10	-2.675995529	DOWN
evm.TU.Contig155_pilon.4	TLN1; talin 1	-2.619487458	DOWN
evm.TU.chr19.430	myosin heavy chain, skeletal muscle, adult-like	-2.465553484	DOWN
evm.TU.chr1.2650	uncharacterized LOC106017522	-2.421370082	DOWN
evm.TU.chr8.9	Tnni3k, Cark, D830019J24Rik; TNNI3 interacting kinase	-2.258016037	DOWN
evm.TU.Contig129_pilon.2	RFX3; regulatory factor X3	-2.064841578	DOWN
evm.TU.chr14.411	uncharacterized LOC106014690	-1.851658038	DOWN
evm.TU.chr1.946	OCA2; OCA2 melanosomal transmembrane protein	-1.752245074	DOWN
evm.TU.chr4.894	CCDC158; LOW QUALITY PROTEIN: coiled-coil domain-containing protein 158	-1.694224875	DOWN

evm.TU.Contig0_pilon.3	DDX25; DEAD-box helicase 25	-1.666559887	DOWN
evm.TU.chr25.244	gastricsin-like	-1.648681152	DOWN
evm.TU.chr10.490	uncharacterized LOC105759503	-1.646033628	DOWN
evm.TU.chr6.310		-1.620773138	DOWN
evm.TU.chr25.88	translocator protein-like	-1.588332186	DOWN
evm.TU.chr11.315	2'-5'-oligoadenylate synthase 1-like	-1.570808116	DOWN
evm.TU.chr21.331	P2RX1; purinergic receptor P2X 1	-1.500443525	DOWN
evm.TU.chr7.412	interferon-induced protein with tetratricopeptide repeats 5	-1.468578316	DOWN
evm.TU.chr14.185	HTR2C; 5-hydroxytryptamine receptor 2C	-1.438224161	DOWN
evm.TU.chr23.310	FABP3; fatty acid binding protein 3	-1.429373762	DOWN
evm.TU.chr7.413	interferon-induced protein with tetratricopeptide repeats 5	-1.419597091	DOWN
evm.TU.chr1.151	PAK1; p21 (RAC1) activated kinase 1	-1.41572738	DOWN
evm.TU.chr3.504	GNG4; G protein subunit gamma 4	-1.298978489	DOWN
evm.TU.chr3.1391	RSAD2, viperin; radical S-adenosyl methionine domain containing 2	-1.291397446	DOWN
evm.TU.chr1.2109	MB; myoglobin	-1.289051507	DOWN
evm.TU.chr13.404		-1.26563184	DOWN
evm.TU.chr13.248	SLC7A9; solute carrier family 7 member 9	-1.260331737	DOWN
evm.TU.Contig153_pilon.7		-1.240533506	DOWN
evm.TU.chr6.426	RACGAP1L; rac GTPase-activating protein 1-like	-1.234969641	DOWN
evm.TU.chr1.150	GDPD4; glycerophosphodiester phosphodiesterase domain containing 4	-1.234614238	DOWN
evm.TU.chr2.536	MARVELD3; MARVEL domain containing 3	-1.222920844	DOWN

evm.TU.chr1.1095	MX; interferon-induced GTP-binding protein Mx-like	-1.221895785	DOWN
evm.TU.chr2.345	INHBA; inhibin beta A subunit	-1.208513785	DOWN
evm.TU.chr4.840	F11, KLKB1; coagulation factor XI	-1.184458899	DOWN
evm.TU.chr2.604	SBK2; SH3 domain binding kinase family member 2	-1.172745175	DOWN
evm.TU.chr8.108	TMEM61; transmembrane protein 61	-1.161839131	DOWN
evm.TU.chr1.442	SHISA2; shisa family member 2	-1.161747959	DOWN
evm.TU.chr27.114		-1.138230815	DOWN
evm.TU.chr3.1390	CMPK2; cytidine/uridine monophosphate kinase 2	-1.108322006	DOWN
evm.TU.chr25.354	LAMB3; laminin subunit beta 3	-1.103511906	DOWN
evm.TU.chr1.1900	FAR2; fatty acyl-CoA reductase 2	-1.080822675	DOWN
evm.TU.chr22.313		-1.073673146	DOWN
evm.TU.chr20.184	LAMC3; laminin subunit gamma 3	-1.051934635	DOWN
evm.TU.chr21.268	C-C motif chemokine 5-like	-1.031432778	DOWN

Supplemental Table S5 Differentially upregulated Genes in Muscovy ducks' muscle tissue

Name	Description	log2FC	trend
evm.TU.chrZ.974	protein FAM151B-like	1.007209111	UP
evm.TU.chr8.679	BCAR3; breast cancer anti-estrogen resistance 3	1.009691154	UP
evm.TU.chr17.122	TNFRSF6B; TNF receptor superfamily member 6b	1.014303282	UP
evm.TU.chr26.173	TOP2A; topoisomerase (DNA) II alpha	1.021598131	UP
evm.TU.chr6.94	ZSWIM2; zinc finger SWIM-type containing 2	1.02589683	UP
evm.TU.chr1.2559	ADAMTS20; ADAM metallopeptidase with thrombospondin type 1 motif 20	1.02954814	UP
evm.TU.chr9.299	VIPR2; vasoactive intestinal peptide receptor 2	1.033216862	UP
evm.TU.chr9.360	CSRNP1; cysteine and serine rich nuclear protein 1	1.040812072	UP
evm.TU.chrZ.835	SNCAIP; synuclein alpha interacting protein	1.046009006	UP
evm.TU.Contig1179_pilon.1	RECQL4; RecQ like helicase 4	1.059420253	UP
evm.TU.chrZ.449	FREM1; FRAS1 related extracellular matrix 1	1.060367288	UP
evm.TU.chr1.2371	APOLD1; apolipoprotein L domain containing 1	1.071804532	UP
evm.TU.chr12.513	PRTG; protogenin	1.076980319	UP
evm.TU.chr9.342	WDR86; WD repeat domain 86	1.084434709	UP
evm.TU.chr7.25	MKI67; marker of proliferation Ki-67	1.097177459	UP
evm.TU.chr3.154	ISM1; isthmin 1	1.099258111	UP
evm.TU.chr10.280	TFRC; transferrin receptor	1.111934946	UP
evm.TU.chr4.750	MARCH1; membrane associated ring-CH-type finger 1	1.117645457	UP
evm.TU.chr1.1733	DYRK4; dual specificity tyrosine phosphorylation regulated kinase 4	1.128874456	UP

evm.TU.chr1.2271	SOCS2; suppressor of cytokine signaling 2	1.129042132	UP
evm.TU.chr1.1499	CD96; CD96 molecule	1.13951661	UP
evm.TU.chr4.321	FAM184B; family with sequence similarity 184 member B	1.146752032	UP
evm.TU.chr25.141	LHFPL5, PMP22A; lipoma HMGIC fusion partner-like 5	1.156115408	UP
evm.TU.chr1.2331	MGP; matrix Gla protein	1.163032442	UP
evm.TU.chr18.98	IGLL1, IGLV, IgL; immunoglobulin lambda-like polypeptide 1	1.166417847	UP
evm.TU.chr7.524	CDK1; cyclin dependent kinase 1	1.176266588	UP
evm.TU.chr1.2448	E2F7; E2F transcription factor 7	1.18041859	UP
evm.TU.chrZ.595	GADD45G; growth arrest and DNA damage inducible gamma	1.185139529	UP
evm.TU.chr14.92	KLHL4; kelch like family member 4	1.194257449	UP
evm.TU.chr17.22	putative iroquois-class homeodomain protein irx-1	1.199402959	UP
evm.TU.chr5.90	WDHD1; WD repeat and HMG-box DNA binding protein 1	1.200960735	UP
evm.TU.chr4.903	JCHAIN, IGJ; joining chain of multimeric IgA and IgM	1.2042309	UP
evm.TU.chr5.1289	INSC; inscuteable homolog (Drosophila)	1.214252787	UP
evm.TU.chrZ.904_evm.TU.chrZ.905	GRIN3A; glutamate ionotropic receptor NMDA type subunit 3A	1.217773583	UP
evm.TU.chrZ.730_evm.TU.chrZ.732	pro-neuregulin-1, membrane-bound isoform-like	1.219307268	UP
evm.TU.chr1.1998	PTN, HB-GAM, HBBM, HBGF-8, HBNF, OSF-1; pleiotrophin	1.221485055	UP
evm.TU.chr2.1032	PAG1; phosphoprotein membrane anchor with glycosphingolipid microdomains 1	1.243184762	UP

evm.TU.chr5.703	KNL1, CASC5; kinetochore scaffold 1	1.249989088	UP
evm.TU.chr1.450	TNFRSF19; TNF receptor superfamily member 19	1.255869282	UP
evm.TU.chr2.928	PENK; proenkephalin	1.261709382	UP
evm.TU.chr4.1109	DKK2; dickkopf WNT signaling pathway inhibitor 2	1.275899159	UP
evm.TU.chr2.545	CDH20; cadherin-20	1.281361055	UP
evm.TU.chr17.160	COL20A1; collagen type XX alpha 1 chain	1.284881333	UP
evm.TU.chr15.473	CCNJL; cyclin J like	1.285804088	UP
evm.TU.chr19.277	urotensin-2 receptor	1.303178516	UP
evm.TU.chr1.1265	LANCL3; LanC like 3	1.316632186	UP
evm.TU.chr16.549	PLK1; polo like kinase 1	1.327353329	UP
evm.TU.chrZ.937	PTGR1; prostaglandin reductase 1	1.327429446	UP
evm.TU.chrZ.998_evm.TU.chrZ.997	ADGRV1, GPR98; adhesion G protein-coupled receptor V1	1.337402763	UP
evm.TU.chr1.2760	SEMA3E; semaphorin 3E	1.341949329	UP
evm.TU.chr5.1201	DBX1; developing brain homeobox 1	1.357285035	UP
evm.TU.chr8.583	RGS1; regulator of G-protein signaling 1	1.359130538	UP
evm.TU.chr12.195	AKAP13; A-kinase anchoring protein 13	1.366671351	UP
evm.TU.chrZ.552	FBP2; fructose-bisphosphatase 2	1.391963749	UP
evm.TU.chr7.35	ADAM12; ADAM metallopeptidase domain 12	1.42893361	UP
evm.TU.chr1.774	METTL21C; methyltransferase like 21C	1.429549183	UP
evm.TU.chr3.1292	TTK; TTK protein kinase	1.432527876	UP

evm.TU.chr1.2524	SRGAP1; SLIT-ROBO Rho GTPase activating protein 1	1.433502421	UP
evm.TU.chr4.448	RASL11B; RAS like family 11 member B	1.448347728	UP
evm.TU.chrZ.623		1.462078935	UP
evm.TU.chr17.152	peroxidasin homolog	1.486434999	UP
evm.TU.chr4.577	MAD2L1; MAD2 mitotic arrest deficient-like 1 (yeast)	1.497846863	UP
evm.TU.chr6.466	SLC15A2; solute carrier family 15 member 2	1.51256504	UP
evm.TU.chr4.943	MTMR7; myotubularin related protein 7	1.51735248	UP
evm.TU.chrZ.141	RXFP3; relaxin/insulin like family peptide receptor 3	1.563925467	UP
evm.TU.chr10.491	PCSK1, PC1; proprotein convertase subtilisin/kexin type 1	1.574489318	UP
evm.TU.chr14.91	KLHL4; kelch like family member 4	1.613598043	UP
evm.TU.chr6.531	MARCO; macrophage receptor with collagenous structure	1.624411963	UP
evm.TU.chrZ.620	SLC27A6; solute carrier family 27 (fatty acid transporter), member 6	1.636559648	UP
evm.TU.chr15.201	GRIA1; glutamate ionotropic receptor AMPA type subunit 1	1.653004654	UP
evm.TU.chr1.2732	LRRC17; leucine rich repeat containing 17	1.665196726	UP
evm.TU.chr1.531	CKAP2; cytoskeleton associated protein 2	1.665385069	UP
evm.TU.chr21.319	COL26A1, EMID2; collagen type XXVI alpha 1 chain	1.782364463	UP
evm.TU.chr1.2026	AKR1D1; aldo-keto reductase family 1 member D1	1.84658794	UP
evm.TU.chr4.131	octopamine receptor-like	1.858681203	UP
evm.TU.chr2.209	NRSN1; neurensin 1	1.861587933	UP
evm.TU.Contig960_pilon.1	LRRC17; leucine-rich repeat-containing protein 17 isoform X1	1.908533822	UP
evm.TU.chrZ.95	SYT4; synaptotagmin 4	1.924256506	UP

evm.TU.chr24.105	B3GAT1; beta-1,3-glucuronyltransferase 1	1.950492156	UP
evm.TU.chr6.658	RPRM; reprimo, TP53 dependent G2 arrest mediator candidate	1.965995624	UP
evm.TU.chr10.287	MASP1; mannan binding lectin serine peptidase 1	1.980502486	UP
evm.TU.chr19.387	CFAP52, WDR16; cilia and flagella associated protein 52	2.087949429	UP
evm.TU.chr4.642	CLGN; calmegin	2.104387799	UP
evm.TU.chr12.419	ISL2, ISLET-2; ISL LIM homeobox 2	2.129421123	UP
evm.TU.chr1.2053	IGF1, IGF-1, IGF-I; insulin like growth factor 1	2.177437275	UP
evm.TU.chrZ.85	SIGLEC15; sialic acid binding Ig like lectin 15	2.17854037	UP
evm.TU.chr10.66	PTX3; pentraxin 3	2.278693185	UP
evm.TU.chr16.218	GSG1L; GSG1 like	2.385889868	UP
evm.TU.chr5.1076	actin, alpha skeletal muscle B-like	2.52945312	UP
evm.TU.chr1.2572	arg8-vasotocin receptor-like	2.591161567	UP
evm.TU.chr2.1088	CDH17; cadherin 17	2.728072151	UP
evm.TU.chr3.661	LRFN2; leucine rich repeat and fibronectin type III domain containing 2	2.829491108	UP
evm.TU.chr12.279	GATM; glycine amidinotransferase	3.053710601	UP
evm.TU.chr15.110_evm.TU.chr15.109	SPOCK1; SPARC/osteonectin, cwcv and kazal like domains proteoglycan 1	3.331401663	UP
evm.TU.chr12.193	synaptic vesicle glycoprotein 2B	3.354329531	UP
evm.TU.chr1.2381_evm.TU.chr1.2382	GRIN2B; glutamate ionotropic receptor NMDA type subunit 2B	3.936848107	UP
evm.TU.chr17.339	cas scaffolding protein family member 4-like	4.159910645	UP

Supplemental Table S6 GO enrichment entries of Muscovy ducks' muscle tissue

Class	GO.ID	Term	P.value
BP	GO:0048856	anatomical structure development	0.04181
BP	GO:0042493	response to drug	0.00015
BP	GO:0051241	negative regulation of multicellular organismal process	0.04243
BP	GO:0050877	nervous system process	0.04878
BP	GO:0048732	gland development	0.02421
BP	GO:0043408	regulation of MAPK cascade	0.02466
BP	GO:0045596	negative regulation of cell differentiation	0.04788
BP	GO:0009617	response to bacterium	0.0104
BP	GO:0001944	vasculature development	0.03407
BP	GO:0048514	blood vessel morphogenesis	0.02164
BP	GO:0031099	regeneration	0.00063
BP	GO:0001666	response to hypoxia	0.00391
BP	GO:0001558	regulation of cell growth	0.04006
BP	GO:0007584	response to nutrient	0.00287
BP	GO:0006816	calcium ion transport	0.01446
BP	GO:0070838	divalent metal ion transport	0.02523
BP	GO:0072511	divalent inorganic cation transport	0.02554
BP	GO:0046677	response to antibiotic	0.02714
BP	GO:0010721	negative regulation of cell development	0.03386

BP	GO:0006163	purine nucleotide metabolic process	0.04668
BP	GO:0048705	skeletal system morphogenesis	0.0278
BP	GO:0097305	response to alcohol	0.03398
BP	GO:0048511	rhythmic process	0.0477
BP	GO:0043901	negative regulation of multi-organism process	0.00764
BP	GO:0032496	response to lipopolysaccharide	0.02538
BP	GO:0002237	response to molecule of bacterial origin	0.03252
BP	GO:0009410	response to xenobiotic stimulus	0.03345
BP	GO:0014823	response to activity	0.00058
BP	GO:0051592	response to calcium ion	0.00325
BP	GO:0045471	response to ethanol	0.02462
BP	GO:0048167	regulation of synaptic plasticity	0.02462
BP	GO:0046034	ATP metabolic process	0.03384
BP	GO:0022612	gland morphogenesis	0.04315
BP	GO:0007204	positive regulation of cytosolic calcium ion concentration	0.04442
BP	GO:0045071	negative regulation of viral genome replication	2.10E-05
BP	GO:0060337	type I interferon signaling pathway	5.50E-05
BP	GO:0010171	body morphogenesis	0.0145
BP	GO:0048678	response to axon injury	0.01641
BP	GO:0043279	response to alkaloid	0.01764
BP	GO:0015718	monocarboxylic acid transport	0.02519

BP	GO:0032355	response to estradiol	0.03522
BP	GO:0007050	cell cycle arrest	0.04587
BP	GO:0002250	adaptive immune response	0.04821
BP	GO:0071359	cellular response to dsRNA	0.00209
BP	GO:0032570	response to progesterone	0.0037
BP	GO:1905809	negative regulation of synapse organization	0.01137
BP	GO:0031100	animal organ regeneration	0.01542
BP	GO:0007492	endoderm development	0.02088
BP	GO:0006970	response to osmotic stress	0.02601
BP	GO:0050974	detection of mechanical stimulus involved in sensory perception	0.0281
BP	GO:0031016	pancreas development	0.02811
BP	GO:0072347	response to anesthetic	0.03835
BP	GO:0007140	male meiotic nuclear division	0.03922
BP	GO:0021510	spinal cord development	0.03922
BP	GO:0097327	response to antineoplastic agent	0.04465
BP	GO:0033273	response to vitamin	0.04559
BP	GO:0055007	cardiac muscle cell differentiation	0.04655
BP	GO:0016321	female meiosis chromosome segregation	0.00047
BP	GO:0060252	positive regulation of glial cell proliferation	0.00056
BP	GO:0035235	ionotropic glutamate receptor signaling pathway	0.0015
BP	GO:0034501	protein localization to kinetochore	0.00188

BP	GO:0051957	positive regulation of amino acid transport	0.00256
BP	GO:0031145	anaphase-promoting complex-dependent catabolic process	0.00309
BP	GO:0043330	response to exogenous dsRNA	0.004
BP	GO:0055023	positive regulation of cardiac muscle tissue growth	0.00626
BP	GO:0007094	mitotic spindle assembly checkpoint	0.00669
BP	GO:0010042	response to manganese ion	0.00669
BP	GO:0003009	skeletal muscle contraction	0.00762
BP	GO:0060416	response to growth hormone	0.00811
BP	GO:0001662	behavioral fear response	0.00969
BP	GO:0002762	negative regulation of myeloid leukocyte differentiation	0.01026
BP	GO:0045740	positive regulation of DNA replication	0.01084
BP	GO:0046688	response to copper ion	0.01206
BP	GO:0014047	glutamate secretion	0.0127
BP	GO:0045428	regulation of nitric oxide biosynthetic process	0.01473
BP	GO:0060323	head morphogenesis	0.01545
BP	GO:0006809	nitric oxide biosynthetic process	0.02016
BP	GO:0048665	neuron fate specification	0.02016
BP	GO:0014075	response to amine	0.02188
BP	GO:0051602	response to electrical stimulus	0.02188
BP	GO:0038034	signal transduction in absence of ligand	0.02368
BP	GO:0097192	extrinsic apoptotic signaling pathway in absence of ligand	0.02368

BP	GO:0046209	nitric oxide metabolic process	0.02462
BP	GO:2001057	reactive nitrogen species metabolic process	0.02557
BP	GO:0015800	acidic amino acid transport	0.02854
BP	GO:0043113	receptor clustering	0.02854
BP	GO:0035094	response to nicotine	0.03061
BP	GO:0072401	signal transduction involved in DNA integrity checkpoint	0.03061
BP	GO:0072422	signal transduction involved in DNA damage checkpoint	0.03061
BP	GO:0072395	signal transduction involved in cell cycle checkpoint	0.03168
BP	GO:0051310	metaphase plate congression	0.035
BP	GO:0019229	regulation of vasoconstriction	0.03731
BP	GO:0045638	negative regulation of myeloid cell differentiation	0.03731
BP	GO:0046148	pigment biosynthetic process	0.03849
BP	GO:0045070	positive regulation of viral genome replication	0.04031
BP	GO:1902106	negative regulation of leukocyte differentiation	0.04091
BP	GO:0071385	cellular response to glucocorticoid stimulus	0.04341
BP	GO:1903426	regulation of reactive oxygen species biosynthetic process	0.04469
BP	GO:0032609	interferon-gamma production	0.04998
BP	GO:0019060	intracellular transport of viral protein in host cell	0.00025
BP	GO:0050689	negative regulation of defense response to virus by host	5.00E-04
BP	GO:0051097	negative regulation of helicase activity	5.00E-04
BP	GO:1902581	multi-organism cellular localization	5.00E-04

BP	GO:0007344	pronuclear fusion	0.00082
BP	GO:0099566	regulation of postsynaptic cytosolic calcium ion concentration	0.00082
BP	GO:0035672	oligopeptide transmembrane transport	0.00226
BP	GO:0048539	bone marrow development	0.00226
BP	GO:0060242	contact inhibition	0.00436
BP	GO:0035457	cellular response to interferon-alpha	0.00611
BP	GO:0035458	cellular response to interferon-beta	0.00709
BP	GO:0050910	detection of mechanical stimulus involved in sensory perception of sound	0.00709
BP	GO:0071073	positive regulation of phospholipid biosynthetic process	0.00813
BP	GO:0007077	mitotic nuclear envelope disassembly	0.00924
BP	GO:0097094	craniofacial suture morphogenesis	0.00924
BP	GO:0001878	response to yeast	0.01041
BP	GO:0014049	positive regulation of glutamate secretion	0.01041
BP	GO:0046689	response to mercury ion	0.01164
BP	GO:0071360	cellular response to exogenous dsRNA	0.01164
BP	GO:0014051	gamma-aminobutyric acid secretion	0.01428
BP	GO:0048266	behavioral response to pain	0.01428
BP	GO:0042538	hyperosmotic salinity response	0.01569
BP	GO:0055012	ventricular cardiac muscle cell differentiation	0.01569
BP	GO:1904666	regulation of ubiquitin protein ligase activity	0.01569
BP	GO:0001553	luteinization	0.01716

BP	GO:0007060	male meiosis chromosome segregation	0.01716
BP	GO:0031017	exocrine pancreas development	0.01716
BP	GO:0061050	regulation of cell growth involved in cardiac muscle cell development	0.01716
BP	GO:0072574	hepatocyte proliferation	0.01716
BP	GO:0072575	epithelial cell proliferation involved in liver morphogenesis	0.01716
BP	GO:0032689	negative regulation of interferon-gamma production	0.01868
BP	GO:0060992	response to fungicide	0.02026
BP	GO:0015812	gamma-aminobutyric acid transport	0.02189
BP	GO:0071305	cellular response to vitamin D	0.02189
BP	GO:0001502	cartilage condensation	0.02358
BP	GO:0033622	integrin activation	0.02358
BP	GO:0045671	negative regulation of osteoclast differentiation	0.02531
BP	GO:0002082	regulation of oxidative phosphorylation	0.0271
BP	GO:0008209	androgen metabolic process	0.0271
BP	GO:0010971	positive regulation of G2/M transition of mitotic cell cycle	0.0271
BP	GO:0098743	cell aggregation	0.0271
BP	GO:0001964	startle response	0.02893
BP	GO:0003094	glomerular filtration	0.02893
BP	GO:0060573	cell fate specification involved in pattern specification	0.02893
BP	GO:0060045	positive regulation of cardiac muscle cell proliferation	0.03082
BP	GO:0060253	negative regulation of glial cell proliferation	0.03082

BP	GO:0072576	liver morphogenesis	0.03082
BP	GO:1902751	positive regulation of cell cycle G2/M phase transition	0.03082
BP	GO:0007390	germ-band shortening	0.03275
BP	GO:0031581	hemidesmosome assembly	0.03275
BP	GO:0051482	positive regulation of cytosolic calcium ion concentration involved in phospholipase C-activating G protein-coupled signaling pathway	0.03275
BP	GO:0014048	regulation of glutamate secretion	0.03472
BP	GO:1904707	positive regulation of vascular associated smooth muscle cell proliferation	0.03472
BP	GO:0006726	eye pigment biosynthetic process	0.03675
BP	GO:0014850	response to muscle activity	0.03675
BP	GO:0032148	activation of protein kinase B activity	0.03675
BP	GO:0035994	response to muscle stretch	0.03881
BP	GO:0042441	eye pigment metabolic process	0.03881
BP	GO:0043324	pigment metabolic process involved in developmental pigmentation	0.03881
BP	GO:0043474	pigment metabolic process involved in pigmentation	0.03881
BP	GO:1903859	regulation of dendrite extension	0.03881
BP	GO:0010738	regulation of protein kinase A signaling	0.04092
BP	GO:0003298	physiological muscle hypertrophy	0.04307
BP	GO:0003301	physiological cardiac muscle hypertrophy	0.04307
BP	GO:0030318	melanocyte differentiation	0.04307
BP	GO:0061049	cell growth involved in cardiac muscle cell development	0.04307

BP	GO:0046164	alcohol catabolic process	0.04527
BP	GO:0097205	renal filtration	0.04527
BP	GO:0030049	muscle filament sliding	0.0475
BP	GO:0008207	C21-steroid hormone metabolic process	0.04978
BP	GO:0042023	DNA endoreduplication	0.04978
BP	GO:0048265	response to pain	0.04978
BP	GO:0050650	chondroitin sulfate proteoglycan biosynthetic process	0.04978
BP	GO:0060325	face morphogenesis	0.04978
BP	GO:0071295	cellular response to vitamin	0.04978
BP	GO:0000705	achiasmate meiosis I	0.00919
BP	GO:0002728	negative regulation of natural killer cell cytokine production	0.00919
BP	GO:0010350	cellular response to magnesium starvation	0.00919
BP	GO:0010430	fatty acid omega-oxidation	0.00919
BP	GO:0015828	tyrosine transport	0.00919
BP	GO:0038130	ERBB4 signaling pathway	0.00919
BP	GO:0045870	positive regulation of single stranded viral RNA replication via double stranded DNA intermediate	0.00919
BP	GO:1901253	negative regulation of intracellular transport of viral material	0.00919
BP	GO:1903096	protein localization to meiotic spindle midzone	0.00919
BP	GO:1904075	positive regulation of trophectodermal cell proliferation	0.00919
BP	GO:1904389	rod bipolar cell differentiation	0.00919
BP	GO:1904391	response to ciliary neurotrophic factor	0.00919

BP	GO:1905463	negative regulation of DNA duplex unwinding	0.00919
BP	GO:1990705	cholangiocyte proliferation	0.00919
BP	GO:2000853	negative regulation of corticosterone secretion	0.00919
BP	GO:0002351	serotonin production involved in inflammatory response	0.0183
BP	GO:0002442	serotonin secretion involved in inflammatory response	0.0183
BP	GO:0002542	Factor XII activation	0.0183
BP	GO:0002554	serotonin secretion by platelet	0.0183
BP	GO:0005986	sucrose biosynthetic process	0.0183
BP	GO:0006233	dTDP biosynthetic process	0.0183
BP	GO:0006601	creatine biosynthetic process	0.0183
BP	GO:0010266	response to vitamin B1	0.0183
BP	GO:0010625	positive regulation of Schwann cell proliferation	0.0183
BP	GO:0014038	regulation of Schwann cell differentiation	0.0183
BP	GO:0015990	electron transport coupled proton transport	0.0183
BP	GO:0031444	slow-twitch skeletal muscle fiber contraction	0.0183
BP	GO:0031583	phospholipase D-activating G protein-coupled receptor signaling pathway	0.0183
BP	GO:0033316	meiotic spindle assembly checkpoint	0.0183
BP	GO:0033626	positive regulation of integrin activation by cell surface receptor linked signal transduction	0.0183
BP	GO:0034155	regulation of toll-like receptor 7 signaling pathway	0.0183
BP	GO:0034157	positive regulation of toll-like receptor 7 signaling pathway	0.0183
BP	GO:0034342	response to type III interferon	0.0183

BP	GO:0035044	sperm aster formation	0.0183
BP	GO:0038129	ERBB3 signaling pathway	0.0183
BP	GO:0044779	meiotic spindle checkpoint	0.0183
BP	GO:0044866	modulation by host of viral exo-alpha-sialidase activity	0.0183
BP	GO:0044867	modulation by host of viral catalytic activity	0.0183
BP	GO:0044869	negative regulation by host of viral exo-alpha-sialidase activity	0.0183
BP	GO:0044870	modulation by host of viral glycoprotein metabolic process	0.0183
BP	GO:0044871	negative regulation by host of viral glycoprotein metabolic process	0.0183
BP	GO:0046072	dTDP metabolic process	0.0183
BP	GO:0048174	negative regulation of short-term neuronal synaptic plasticity	0.0183
BP	GO:0048600	oocyte fate commitment	0.0183
BP	GO:0051867	general adaptation syndrome, behavioral process	0.0183
BP	GO:0052403	negative regulation by host of symbiont catalytic activity	0.0183
BP	GO:0061782	vesicle fusion with vesicle	0.0183
BP	GO:0070060	'de novo' actin filament nucleation	0.0183
BP	GO:1902488	cholangiocyte apoptotic process	0.0183
BP	GO:1903015	regulation of exo-alpha-sialidase activity	0.0183
BP	GO:1903016	negative regulation of exo-alpha-sialidase activity	0.0183
BP	GO:1904192	regulation of cholangiocyte apoptotic process	0.0183
BP	GO:1904193	negative regulation of cholangiocyte apoptotic process	0.0183
BP	GO:1904644	cellular response to curcumin	0.0183

BP	GO:1905414	negative regulation of dense core granule exocytosis	0.0183
BP	GO:1905415	positive regulation of dense core granule exocytosis	0.0183
BP	GO:1905432	regulation of retrograde trans-synaptic signaling by neuropeptide	0.0183
BP	GO:1905433	negative regulation of retrograde trans-synaptic signaling by neuropeptide	0.0183
BP	GO:0002254	kinin cascade	0.02732
BP	GO:0002314	germinal center B cell differentiation	0.02732
BP	GO:0002353	plasma kallikrein-kinin cascade	0.02732
BP	GO:0002541	activation of plasma proteins involved in acute inflammatory response	0.02732
BP	GO:0005985	sucrose metabolic process	0.02732
BP	GO:0006227	dUDP biosynthetic process	0.02732
BP	GO:0006235	dTTP biosynthetic process	0.02732
BP	GO:0007058	spindle assembly involved in female meiosis II	0.02732
BP	GO:0007284	spermatogonial cell division	0.02732
BP	GO:0009139	pyrimidine nucleoside diphosphate biosynthetic process	0.02732
BP	GO:0009196	pyrimidine deoxyribonucleoside diphosphate metabolic process	0.02732
BP	GO:0009197	pyrimidine deoxyribonucleoside diphosphate biosynthetic process	0.02732
BP	GO:0009212	pyrimidine deoxyribonucleoside triphosphate biosynthetic process	0.02732
BP	GO:0009441	glycolate metabolic process	0.02732
BP	GO:0010025	wax biosynthetic process	0.02732
BP	GO:0010157	response to chlorate	0.02732
BP	GO:0010166	wax metabolic process	0.02732

BP	GO:0021650	vestibulocochlear nerve formation	0.02732
BP	GO:0030726	male germline ring canal formation	0.02732
BP	GO:0030954	astral microtubule nucleation	0.02732
BP	GO:0033590	response to cobalamin	0.02732
BP	GO:0034165	positive regulation of toll-like receptor 9 signaling pathway	0.02732
BP	GO:0035442	dipeptide transmembrane transport	0.02732
BP	GO:0036292	DNA rewinding	0.02732
BP	GO:0042938	dipeptide transport	0.02732
BP	GO:0043060	meiotic metaphase I plate congression	0.02732
BP	GO:0044868	modulation by host of viral molecular function	0.02732
BP	GO:0046075	dTTP metabolic process	0.02732
BP	GO:0046077	dUDP metabolic process	0.02732
BP	GO:0051311	meiotic metaphase plate congression	0.02732
BP	GO:0051866	general adaptation syndrome	0.02732
BP	GO:0051919	positive regulation of fibrinolysis	0.02732
BP	GO:0052199	negative regulation of catalytic activity in other organism involved in symbiotic interaction	0.02732
BP	GO:0052422	modulation by host of symbiont catalytic activity	0.02732
BP	GO:0071386	cellular response to corticosterone stimulus	0.02732
BP	GO:0071930	negative regulation of transcription involved in G1/S transition of mitotic cell cycle	0.02732
BP	GO:0099082	retrograde trans-synaptic signaling by neuropeptide	0.02732
BP	GO:1901994	negative regulation of meiotic cell cycle phase transition	0.02732

BP	GO:1902103	negative regulation of metaphase/anaphase transition of meiotic cell cycle	0.02732
BP	GO:1902951	negative regulation of dendritic spine maintenance	0.02732
BP	GO:1903407	negative regulation of sodium:potassium-exchanging ATPase activity	0.02732
BP	GO:1903408	positive regulation of sodium:potassium-exchanging ATPase activity	0.02732
BP	GO:1905133	negative regulation of meiotic chromosome separation	0.02732
BP	GO:1905460	negative regulation of vascular associated smooth muscle cell apoptotic process	0.02732
BP	GO:2001245	regulation of phosphatidylcholine biosynthetic process	0.02732
BP	GO:0003010	voluntary skeletal muscle contraction	0.03626
BP	GO:0007208	phospholipase C-activating serotonin receptor signaling pathway	0.03626
BP	GO:0009211	pyrimidine deoxyribonucleoside triphosphate metabolic process	0.03626
BP	GO:0014721	twitch skeletal muscle contraction	0.03626
BP	GO:0015811	L-cystine transport	0.03626
BP	GO:0021773	striatal medium spiny neuron differentiation	0.03626
BP	GO:0030263	apoptotic chromosome condensation	0.03626
BP	GO:0035476	angioblast cell migration	0.03626
BP	GO:0035962	response to interleukin-13	0.03626
BP	GO:0045636	positive regulation of melanocyte differentiation	0.03626
BP	GO:0046005	positive regulation of circadian sleep/wake cycle, REM sleep	0.03626
BP	GO:0046351	disaccharide biosynthetic process	0.03626
BP	GO:0052203	modulation of catalytic activity in other organism involved in symbiotic interaction	0.03626
BP	GO:0060741	prostate gland stromal morphogenesis	0.03626

BP	GO:0061534	gamma-aminobutyric acid secretion, neurotransmission	0.03626
BP	GO:0070194	synaptonemal complex disassembly	0.03626
BP	GO:0071418	cellular response to amine stimulus	0.03626
BP	GO:0072560	type B pancreatic cell maturation	0.03626
BP	GO:0099538	synaptic signaling via neuropeptide	0.03626
BP	GO:0099540	trans-synaptic signaling by neuropeptide	0.03626
BP	GO:0150172	regulation of phosphatidylcholine metabolic process	0.03626
BP	GO:1900141	regulation of oligodendrocyte apoptotic process	0.03626
BP	GO:1900142	negative regulation of oligodendrocyte apoptotic process	0.03626
BP	GO:1904395	positive regulation of skeletal muscle acetylcholine-gated channel clustering	0.03626
BP	GO:1904643	response to curcumin	0.03626
BP	GO:1905448	positive regulation of mitochondrial ATP synthesis coupled electron transport	0.03626
BP	GO:2000078	positive regulation of type B pancreatic cell development	0.03626
BP	GO:0002071	glandular epithelial cell maturation	0.04512
BP	GO:0009189	deoxyribonucleoside diphosphate biosynthetic process	0.04512
BP	GO:0014834	skeletal muscle satellite cell maintenance involved in skeletal muscle regeneration	0.04512
BP	GO:0021648	vestibulocochlear nerve morphogenesis	0.04512
BP	GO:0043396	corticotropin-releasing hormone secretion	0.04512
BP	GO:0043397	regulation of corticotropin-releasing hormone secretion	0.04512
BP	GO:0045578	negative regulation of B cell differentiation	0.04512
BP	GO:0045914	negative regulation of catecholamine metabolic process	0.04512

BP	GO:0045963	negative regulation of dopamine metabolic process	0.04512
BP	GO:0046882	negative regulation of follicle-stimulating hormone secretion	0.04512
BP	GO:0046960	sensitization	0.04512
BP	GO:0051309	female meiosis chromosome separation	0.04512
BP	GO:0051799	negative regulation of hair follicle development	0.04512
BP	GO:0052405	negative regulation by host of symbiont molecular function	0.04512
BP	GO:0060718	chorionic trophoblast cell differentiation	0.04512
BP	GO:0061554	ganglion formation	0.04512
BP	GO:0061559	cranial ganglion morphogenesis	0.04512
BP	GO:0061560	cranial ganglion formation	0.04512
BP	GO:0061737	leukotriene signaling pathway	0.04512
BP	GO:0070829	heterochromatin maintenance	0.04512
BP	GO:0072656	maintenance of protein location in mitochondrion	0.04512
BP	GO:0098989	NMDA selective glutamate receptor signaling pathway	0.04512
BP	GO:1903367	positive regulation of fear response	0.04512
BP	GO:1903406	regulation of sodium:potassium-exchanging ATPase activity	0.04512
BP	GO:1904373	response to kainic acid	0.04512
BP	GO:1904393	regulation of skeletal muscle acetylcholine-gated channel clustering	0.04512
BP	GO:1990418	response to insulin-like growth factor stimulus	0.04512
BP	GO:2000987	positive regulation of behavioral fear response	0.04512
CC	GO:0005604	basement membrane	0.00019

CC	GO:0005615	extracellular space	0.00025
CC	GO:0000942	condensed nuclear chromosome outer kinetochore	8.00E-04
CC	GO:0099061	integral component of postsynaptic density membrane	0.00221
CC	GO:0070382	exocytic vesicle	0.00497
CC	GO:0017146	NMDA selective glutamate receptor complex	0.00508
CC	GO:0031594	neuromuscular junction	0.00595
CC	GO:0043509	activin A complex	0.00908
CC	GO:0071748	monomeric IgA immunoglobulin complex	0.00908
CC	GO:0071752	secretory dimeric IgA immunoglobulin complex	0.00908
CC	GO:0071756	pentameric IgM immunoglobulin complex	0.00908
CC	GO:0032982	myosin filament	0.01017
CC	GO:0044306	neuron projection terminus	0.0154
CC	GO:0009330	DNA topoisomerase type II (double strand cut, ATP-hydrolyzing) complex	0.01807
CC	GO:0043512	inhibin A complex	0.01807
CC	GO:0097125	cyclin B1-CDK1 complex	0.01807
CC	GO:1990742	microvesicle	0.01807
CC	GO:0005859	muscle myosin complex	0.0198
CC	GO:0014704	intercalated disc	0.02384
CC	GO:0005610	laminin-5 complex	0.02699
CC	GO:0016942	insulin-like growth factor binding protein complex	0.02699
CC	GO:0036454	growth factor complex	0.02699

CC	GO:0042567	insulin-like growth factor ternary complex	0.02699
CC	GO:0043511	inhibin complex	0.02699
CC	GO:0044308	axonal spine	0.02699
CC	GO:1990696	USH2 complex	0.02699
CC	GO:0016460	myosin II complex	0.03012
CC	GO:0043679	axon terminus	0.03423
CC	GO:0002141	stereocilia ankle link	0.03582
CC	GO:0002142	stereocilia ankle link complex	0.03582
CC	GO:0035867	alphav-beta3 integrin-IGF-1-IGF1R complex	0.03582
CC	GO:0008021	synaptic vesicle	0.03823
CC	GO:0030017	sarcomere	0.03926
CC	GO:0098984	neuron to neuron synapse	0.04007
CC	GO:0097060	synaptic membrane	0.04065
CC	GO:0032127	dense core granule membrane	0.04458
CC	GO:1990712	HFE-transferrin receptor complex	0.04458
CC	GO:0098793	presynapse	0.04509
CC	GO:0031301	integral component of organelle membrane	0.04931
MF	GO:0005427	proton-dependent oligopeptide secondary active transmembrane transporter activity	0.00023
MF	GO:0099583	neurotransmitter receptor activity involved in regulation of postsynaptic cytosolic calcium ion concentration	0.00045
MF	GO:0004972	NMDA glutamate receptor activity	0.00112

MF	GO:0016594	glycine binding	0.00328
MF	GO:0017018	myosin phosphatase activity	0.00328
MF	GO:0003725	double-stranded RNA binding	0.00365
MF	GO:0005231	excitatory extracellular ligand-gated ion channel activity	0.00477
MF	GO:0008144	drug binding	0.00487
MF	GO:0008083	growth factor activity	0.00686
MF	GO:0001587	Gq/11-coupled serotonin receptor activity	0.00878
MF	GO:0001604	urotensin II receptor activity	0.00878
MF	GO:0001730	2'-5'-oligoadenylate synthetase activity	0.00878
MF	GO:0001872	(1->3)-beta-D-glucan binding	0.00878
MF	GO:0004998	transferrin receptor activity	0.00878
MF	GO:0005302	L-tyrosine transmembrane transporter activity	0.00878
MF	GO:0015068	glycine amidinotransferase activity	0.00878
MF	GO:0015334	high-affinity oligopeptide transmembrane transporter activity	0.00878
MF	GO:0036185	13-lipoxin reductase activity	0.00878
MF	GO:0042302	structural constituent of cuticle	0.00878
MF	GO:0047568	3-oxo-5-beta-steroid 4-dehydrogenase activity	0.00878
MF	GO:0071916	dipeptide transmembrane transporter activity	0.00878
MF	GO:0097257	leukotriene B4 12-hydroxy dehydrogenase activity	0.00878
MF	GO:0042165	neurotransmitter binding	0.00955
MF	GO:0005031	tumor necrosis factor-activated receptor activity	0.01066

MF	GO:0005035	death receptor activity	0.01066
MF	GO:0099529	neurotransmitter receptor activity involved in regulation of postsynaptic membrane potential	0.01066
MF	GO:0000339	RNA cap binding	0.01184
MF	GO:0005159	insulin-like growth factor receptor binding	0.01184
MF	GO:0005524	ATP binding	0.01294
MF	GO:0035639	purine ribonucleoside triphosphate binding	0.01464
MF	GO:0004798	thymidylate kinase activity	0.01749
MF	GO:0008117	sphinganine-1-phosphate aldolase activity	0.01749
MF	GO:0008147	structural constituent of bone	0.01749
MF	GO:0008269	JAK pathway signal transduction adaptor activity	0.01749
MF	GO:0019862	IgA binding	0.01749
MF	GO:0031728	CCR3 chemokine receptor binding	0.01749
MF	GO:0033862	UMP kinase activity	0.01749
MF	GO:0042132	fructose 1,6-bisphosphate 1-phosphatase activity	0.01749
MF	GO:0071886	1-(4-iodo-2,5-dimethoxyphenyl)propan-2-amine binding	0.01749
MF	GO:0048365	Rac GTPase binding	0.02017
MF	GO:0030332	cyclin binding	0.02323
MF	GO:0005179	hormone activity	0.02354
MF	GO:0003918	DNA topoisomerase type II (double strand cut, ATP-hydrolyzing) activity	0.02612
MF	GO:0004999	vasoactive intestinal polypeptide receptor activity	0.02612
MF	GO:0005497	androgen binding	0.02612

MF	GO:0009041	uridylate kinase activity	0.02612
MF	GO:0015333	peptide:proton symporter activity	0.02612
MF	GO:0030348	syntaxin-3 binding	0.02612
MF	GO:0035373	chondroitin sulfate proteoglycan binding	0.02612
MF	GO:0080019	fatty-acyl-CoA reductase (alcohol-forming) activity	0.02612
MF	GO:1904399	heparan sulfate binding	0.02612
MF	GO:0017022	myosin binding	0.02812
MF	GO:0008266	poly(U) RNA binding	0.03009
MF	GO:0032027	myosin light chain binding	0.03009
MF	GO:0032559	adenyl ribonucleotide binding	0.03084
MF	GO:0005126	cytokine receptor binding	0.03189
MF	GO:0004127	cytidylate kinase activity	0.03467
MF	GO:0004971	AMPA glutamate receptor activity	0.03467
MF	GO:0005131	growth hormone receptor binding	0.03467
MF	GO:0010997	anaphase-promoting complex binding	0.03467
MF	GO:0015018	galactosylgalactosylxylosylprotein 3-beta-glucuronosyltransferase activity	0.03467
MF	GO:0015184	L-cystine transmembrane transporter activity	0.03467
MF	GO:0050062	long-chain-fatty-acyl-CoA reductase activity	0.03467
MF	GO:0061821	telomeric D-loop binding	0.03467
MF	GO:0062037	D-loop DNA binding	0.03467
MF	GO:0070009	serine-type aminopeptidase activity	0.03467

MF	GO:0070699	type II activin receptor binding	0.03467
MF	GO:0032555	purine ribonucleotide binding	0.03508
MF	GO:0030554	adenyl nucleotide binding	0.03517
MF	GO:0005496	steroid binding	0.03643
MF	GO:0044877	protein-containing complex binding	0.03714
MF	GO:0000146	microfilament motor activity	0.03764
MF	GO:0003823	antigen binding	0.03764
MF	GO:0032553	ribonucleotide binding	0.03938
MF	GO:0017076	purine nucleotide binding	0.0409
MF	GO:0005178	integrin binding	0.04294
MF	GO:0004931	extracellularly ATP-gated cation channel activity	0.04315
MF	GO:0038085	vascular endothelial growth factor binding	0.04315
MF	GO:0070538	oleic acid binding	0.04315
MF	GO:0001965	G-protein alpha-subunit binding	0.04798

Supplemental Table S7 Primer information

Gene	Primer Sequence	GenBank Number
AMPK-α	F: CGGCAAAGTCAAGGTTGGCAAAC<br>R: TCCTACAACATCAAGGCTGCGAATC	XM_027447031.2
CPT-1A	F: CCGCCATCTGTTCTGCCTCTATG<br>R: TGTGTTGCTGTGGTGTCTGACTTG	XM_027457809.2
ACC1	F: CACAGATCCAGAGCACAGCACTTC<br>R: GGCAGGCAGTATCCGTTCATCAC	XM_038165892.1
CD36	F: TCGTTTCGCAGTTCCTCGTGAAG<br>R: AGCTGTTGTGCAGTTCTGGGATATG	XM_038183702.1
LPL	F: TGGACATTGGTGACCTGCTTATGC<br>R: TCGCCTGACTTCACTCTGACTCTC	XM_027446391.2
IGF1	F: CTTTGGGAGTTGTGGGTGGAAAC<br>R: GTAGTCTTGTGGTTGTGGAGTACAG	XM_038187451.1
VIPR2	F: GGAGGAAATGACCAGTCACAGT<br>R: CGGAAGTGCGTTCAGGAAAC	XM_038174746.1
AMH	F: CCAGGCAAGGCTGTGGTTTA<br>R: ACTGGGTGTCCCTAGTGAGG	NM_001310362.1
GRIN3A	F: TCCGACAGCACCAAGACATC<br>R: CTCTGTGAGCTCTTGCGTCA	XM_038170310.1
PAK1	F: CCTCGGTGTCTGAAGACGAA<br>R: TGGCCACATCTCTGGTAGGA	XM_038177853.1
SSTR2	F: GCATGTTCGACTTCGTCGTG<br>R: TCTCGTTGAGCCTGGACTTG	XM_038165592.1
GADPH	F: GGTAGTGAAGGCTGCTGCTGATG<br>R: GGAGGAATGGCTGTCACCGTTG	XM_038180584.1
